# The Numbers Tell It All: Students Don't Like Numbers!

**DOI:** 10.1371/journal.pone.0083443

**Published:** 2013-12-16

**Authors:** Bob Uttl, Carmela A. White, Alain Morin

**Affiliations:** Department of Psychology, Mount Royal University, Calgary, Alberta, Canada; Universita` degli Studi di Milano (University of Milan), Italy

## Abstract

Undergraduate Students' interest in taking quantitative vs. non quantitative courses has received limited attention even though it has important consequences for higher education. Previous studies have collected course interest ratings at the end of the courses as part of student evaluation of teaching (SET) ratings, which may confound prior interest in taking these courses with students' actual experience in taking them. This study is the first to examine undergraduate students' interest in quantitative vs. non quantitative courses in their first year of studies before they have taken any quantitative courses. Three hundred and forty students were presented with descriptions of 44 psychology courses and asked to rate their interest in taking each course. Student interest in taking quantitative vs non quantitative courses was very low; the mean interest in statistics courses was nearly 6 *SD*s below the mean interest in non quantitative courses. Moreover, women were less interested in taking quantitative courses than men. Our findings have several far-reaching implications. First, evaluating professors teaching quantitative vs. non quantitative courses against the same SET standard may be inappropriate. Second, if the same SET standard is used for the evaluation of faculty teaching quantitative vs. non quantitative courses, faculty are likely to teach to SETs rather than focus on student learning. Third, universities interested primarily in student satisfaction may want to expunge quantitative courses from their curricula. In contrast, universities interested in student learning may want to abandon SETs as a primary measure of faculty teaching effectiveness. Fourth, undergraduate students who are not interested in taking quantitative courses are unlikely to pursue graduate studies in quantitative psychology and unlikely to be able to competently analyze data independently.

## Introduction

Are psychology and other undergraduate students less interested in taking quantitative courses such as statistics and research methods courses relative to other non quantitative courses? If so, are these differences small and perhaps ignorable, or are they large enough to require substantive changes in higher education? Student interest or lack of interest in taking quantitative vs. non quantitative courses has important implications for at least four aspects of higher education: evaluation of faculty teaching effectiveness, student learning outcomes, balance of institutional focus on student learning vs. student satisfaction, student success following graduation (e.g., gaining admission to graduate schools, finding paid employment in their field), and even the survival of psychology and other fields as science [1].

 The mission statements of colleges and universities often focus on two goals: student learning/program outcomes and student satisfaction. Consistent with the latter goal, nearly all colleges and universities in Canada and USA employ anonymous student evaluation of teaching (SET) surveys to measure student satisfaction and evaluate “teaching effectiveness” of their faculty. If students are much less interested in quantitative vs. non quantitative courses, faculty teaching quantitative courses may be under greater pressure to focus on student satisfaction as measured by their SETs rather than on student learning. In contrast, evaluation of student learning and program outcomes are rare, primarily affecting only those professional programs where graduates need to pass various professional exams to enter their professions (e.g., Canadian Registered Nurse Examination or CRNE). In turn, the scarcity of evaluation of student learning likely fuels faculty members' focus on student satisfaction rather than on learning.

 Although only a limited number of studies have examined the relationship between student course interest and SET ratings, they suggests that students' prior course interest is one of the strongest predictor of SET ratings [2–4]. Previous research has also suggested that students are less interested in taking quantitative vs. non quantitative courses [3], and that quantitative courses receive lower SET ratings than non quantitative courses [3]. Accordingly, some experts [5,6] concluded that SET ratings require an adjustment for student interest and [3] suggested that SET ratings in quantitative courses may require an adjustment to account for students' poor interest and motivation to take these courses. To our knowledge, however, no prior studies have examined student interest in taking quantitative vs. non quantitative courses taught within a single discipline such as psychology, where differences in student interest in taking quantitative vs. non quantitative courses may be even larger than the differences reported previously [3] across a wide range of disciplines. Moreover, previous studies that examined student interest in quantitative vs. non quantitative courses collected student interest ratings retrospectively, at the time of SETs. However, such retrospective assessment may be influenced by students' experience in these courses rather than only by their prior course interest.

 A lack of interest in taking quantitative courses is also likely to limit students' career choices. For example, a recent survey of various psychology programs showed that courses with quantitative content – statistics and research methods courses – were the most frequently required courses by all programs within psychology (i.e., clinical/counseling, educational/school, industrial/organizational, experimental, mixed) [7]. Similarly, quantitative courses such as statistics, research methods, and psychometrics are required or strongly preferred for many of the higher-paying jobs that require only a BA/BSc degrees (e.g., psychometrists in some jurisdictions).

 Moreover, concerns about the shortage of quantitative psychologists and the quantitative training in doctoral programs in all areas of psychology have led the APA to commission the *Task Force for Increasing the Number of Quantitative Psychologists* [8]. Among its many observations, the Task Force noted that graduate quantitative psychology programs were not receiving enough applications and not graduating enough doctoral candidates to meet market demands. When does this lack of interest in quantitative methods first begin? Anecdotal evidence suggests that students entering psychology at the undergraduate level already view quantitative courses not with interest but as a necessary evil. However, to our knowledge, no prior study has systematically examined undergraduate student interests in quantitative vs. non quantitative courses.

Finally, women vs. men are less likely to participate in and pursue careers in mathematically intensive science, technology, engineering, and mathematics (STEM) fields [9]. In a comprehensive, consensus review of the existing literature on sex differences in science and mathematics, Halpern and colleagues [9] concluded that there are many different factors contributing to sex differences in math and science, including early experiences, biological factors, educational policies and culture. Thus, women vs. men may be less interested in taking quantitative university courses whereas there may be no or only minimal differences between women and men's interest in taking other, non-quantitative courses. If so, professors teaching quantitative courses within disciplines that draw primarily women (e.g., psychology) may be facing especially uninterested students.

 Accordingly, our study had four major objectives. The first objective was to examine student interest in taking quantitative vs. non quantitative psychology courses within a single discipline and prior to having taken them. The second objective was to determine the size of any differences in student interest in taking quantitative vs. non quantitative courses. Previous studies [3] reported that students were less interested in taking quantitative vs. non quantitative courses but they did not report the size of these differences (i.e., effect size measures) nor sufficient information (e.g., *SDs*) to calculate them. The third objective was to examine whether quantitative vs. non quantitative psychology courses are rated differently by students majoring in psychology vs. other fields. Finally, we also examined whether women vs. men are less interested in taking quantitative vs. non-quantitative courses.

## Methods

### Ethics Statement

 The study was approved by Mount Royal University Human Research Ethics Board and all participants gave written consent to participate in the study. 

### Participants

Participants were 340 undergraduate students (mean age = 22.1 years, range = 17 to 57 years; 77.6% females, 22.4% males) enrolled in introductory psychology courses at Mount Royal University, Calgary, Canada. English was the first language of 81.1% participants. Fifty one participants were majoring in psychology and 289 were majoring in other fields or had not yet declared their major. 

### Materials

 The course interest survey consisted of 44 titles and descriptions of all psychology courses offered in the 2012-2013 Mount Royal University calendar except the two introductory psychology first year courses participants were registered in. Next to each course description was the course interest rating scale. The 5-point rating scale ranged from 1 = *Not at all interested* to 5 = *Very interested*.

 The courses were classified as having a high (3 courses), moderate (6 courses) or low (34 courses) amount of quantitative content (QC). The three courses with high QC were statistics courses: PSYC 2210 Statistical Methods for Psychology I, PSYC 2211 Statistical Methods for Psychology II, and PSYC 4412 Advanced Statistical Methods for Psychology. The six courses with moderate QC were primarily research methods and research intensive courses: PSYC 2213 Research Methods I, PSYC 4413 Research Methods II, PSYC 4405 Psychometrics, PSYC 4476 Research Methods in Brain and Behavior, PSYC 3199/4199 Directed Reading, PSYC 5110 Honours Seminar I, and PSYC 5120 Honours Seminar II. The remaining 34 courses with minimal or no (low QC) were 33 courses focusing on various content areas within psychology itself and one course focusing on history of psychology rather than on any particular content area (PSYC 3305 History of Psychological Thought). Finally, one course, PSYC 3199/4199 Directed Reading, was unclassified as it could involve a range of activities (e.g., conducting a literature review, a small research project including data collection and analysis) and include moderate to no quantitative content.

 Two high QC courses (PSYC 2210 and PSYC 2211) and one moderate QC course (PSYC 2213) are required courses for all psychology majors. Another high QC course (PSYC 4412) and three moderate QC courses (PSYC 4413, PSYC 5110, PSYC 5120) are required courses for all psychology students graduating with an Honours degree.

### Procedure

As part of a larger study lasting 1.5 to 2 hours, participants (tested in small groups) were presented with the course interest survey and asked to rate how interested they were in taking each course based on its description and their interests, regardless of whether the course may be required for their degree. In addition, participants were asked to complete a basic demographic questionnaire (e.g., age, sex, whether English was their first language) and to state their study major.

## Results

**Figure 1:**
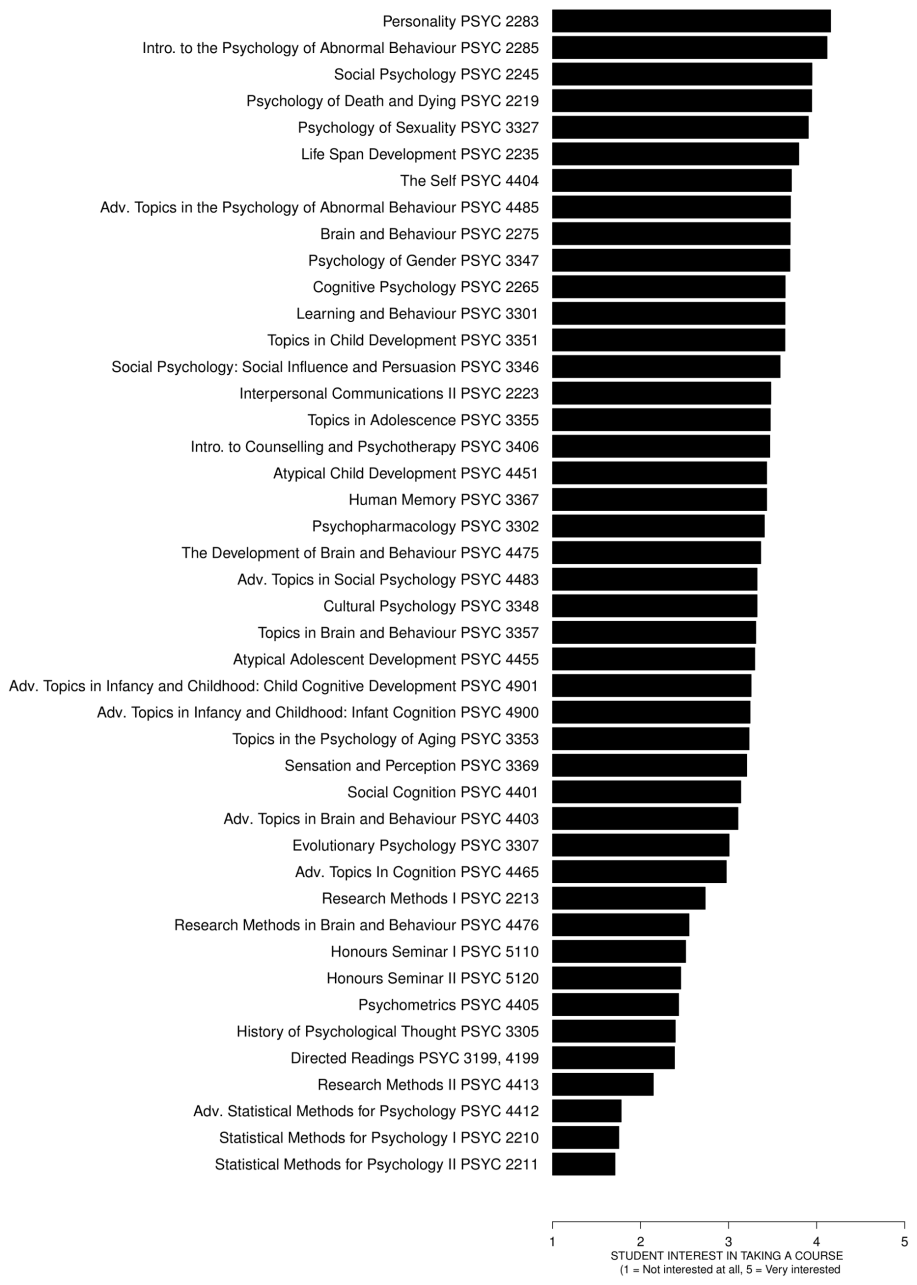
The mean interest ratings for all courses. The courses are ordered from the highest rated to the lowest rated courses. The courses with high QC (statistics courses) were rated the lowest, the courses with moderate QC (research methods and research intensive courses) were rated somewhat higher, and the courses with low QC were rated the highest, with the exception of the History of Psychological Thought course.

Figure 1 shows the interest ratings for all courses, ordered from the highest rated to the lowest rated courses. The courses with high QC (statistics courses) were rated the lowest, the courses with moderate QC (research methods and research intensive courses) were rated somewhat higher, and the courses with low QC were rated the highest, with the exception of the History of Psychological Thought course. The top five highest rated courses focused on personality, abnormal behavior, death and dying, social psychology, and sexuality.

**Figure 2:**
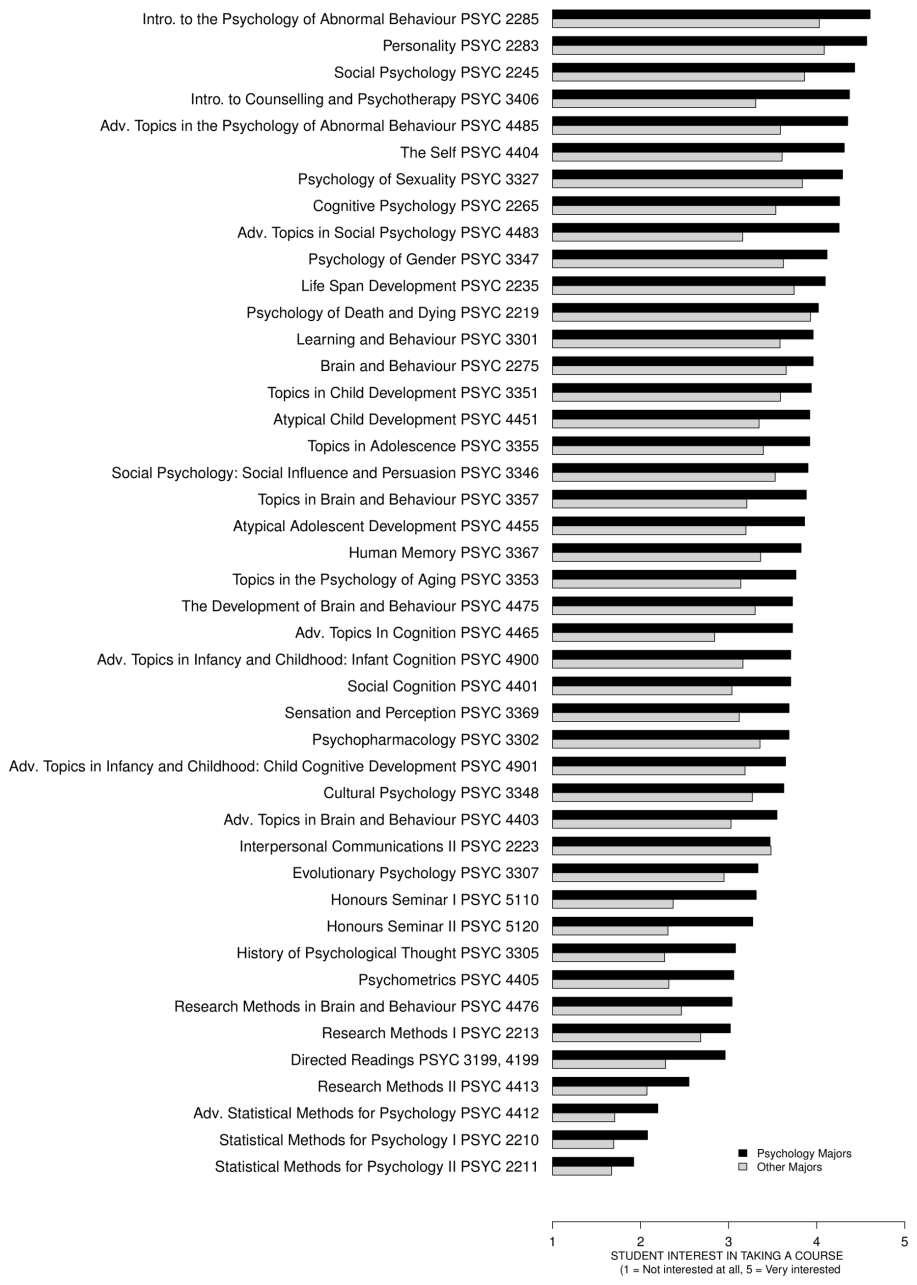
The mean interest ratings for all courses for psychology vs. other majors. The courses are ordered from the highest rated to the lowest rated courses using psychology majors' ratings. Overall, psychology majors expressed more interest in taking various courses than other majors. More importantly, regardless of the major, high QC courses were rated the lowest, moderate QC courses were rated somewhat higher, and low QC courses were rated the highest.

Figure 2 shows the interest ratings for all courses for psychology vs. other majors, ordered from the highest rated to the lowest rated courses using psychology majors' ratings. Overall, psychology majors expressed more interest in taking various courses than other majors. More importantly, regardless of the major, high QC courses were rated the lowest, moderate QC courses were rated somewhat higher, and low QC courses were rated the highest. Students majoring in psychology were most interested in taking courses focusing on abnormal behavior, personality, social psychology, and counselling and psychotherapy.

**Figure 3:**
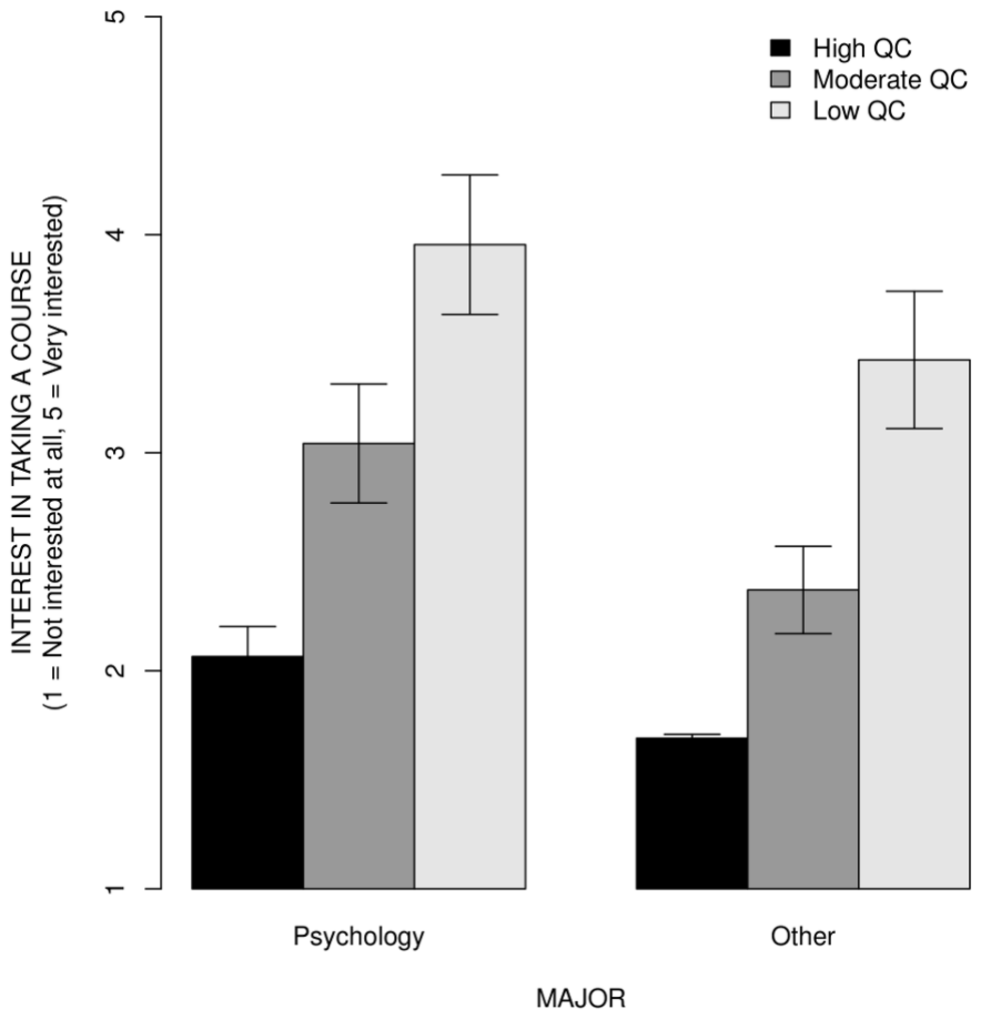
The mean course interest ratings by quantitative content, for psychology vs. other majors. The error bars indicate ± 1 *SD* to facilitate visualization of the effect size. High QC courses were rated nearly 6 *SD* below low QC courses by both psychology majors and other majors, using low QC courses *SD* as a reference. Moderate QC courses were rated approximately 3 *SD* below low QC courses by both psychology and other majors majors, using low QC courses *SD* as a reference. Psychology majors were more interested in taking all three types of courses than other majors.

Figure 3 shows the mean of average course interest ratings for courses with high, moderate, and low QC, for psychology vs. other majors, with error bars indicating ± 1 *SD* to facilitate visualization of the effect size. High QC courses were rated more than 5 *SD* below low QC courses by both psychology majors (5.91 *SD*) and other majors (5.51 *SD*), using low QC courses SD as a reference SD. Moderate QC courses were rated approximately 3 *SD* below low QC courses by both psychology (2.85 *SD*) and other majors (3.35 *SD*), using low QC courses SD as a reference SD. However, psychology majors were more interested in taking all three types of courses than other majors. These observations were confirmed by two ANOVAs. The ANOVA on the mean course ratings with major (psychology, not psychology) and course type (low QC, medium QC, high QC) as between subject factors revealed a significant main effect of major, *F*(1,40) = 216.41, MSe = 0.028, *p* < .001 and a significant main effect of course type, *F*(1,40) = 165.71, MSe = 0.152, *p* < .001. A major by course type interaction was not significant, *F*(1,40) = 3.45, MSe = 0.028, *p* = .854. Similarly, the ANOVA on participants' data with major (psychology, not psychology) as a between subject factor and course type (low QC, medium QC, high QC) as within subject factor revealed a significant main effect of major, *F*(1,338) = 25.24, MSe = 1.43, *p* < .001; a significant main effect of course type, *F*(1,676) = 688.96, MSe = 0.37, *p* < .001; and only a marginally significant major by course type interaction, *F*(1,676) = 2.59, MSe = 0.37, *p* = .076.

**Figure 4:**
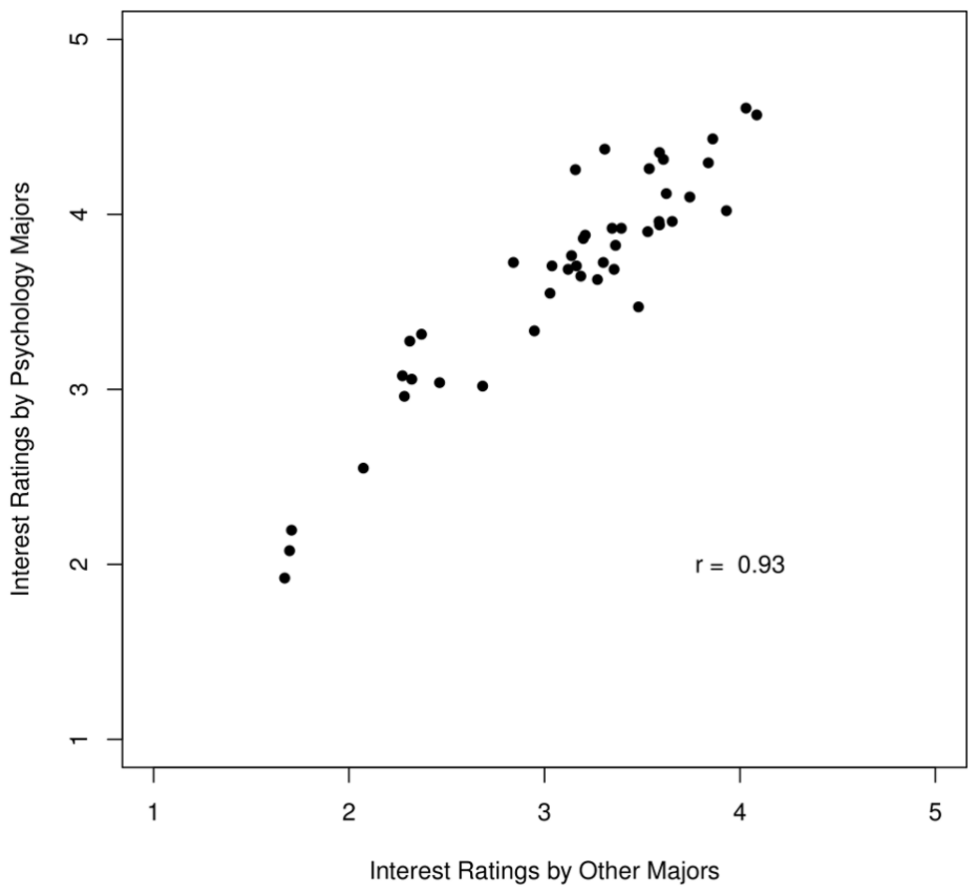
The scatterplot of mean course interest ratings given by psychology and other majors. The figure highlights that the ratings provided by the two groups of students were highly correlated.

Figure 4 shows a scatterplot of mean course interest ratings given by psychology and other majors. The figure highlights that the ratings provided by the two groups of students were highly correlated, *r*(42) = .93, *95% CI* = (.88, .96).

**Figure 5:**
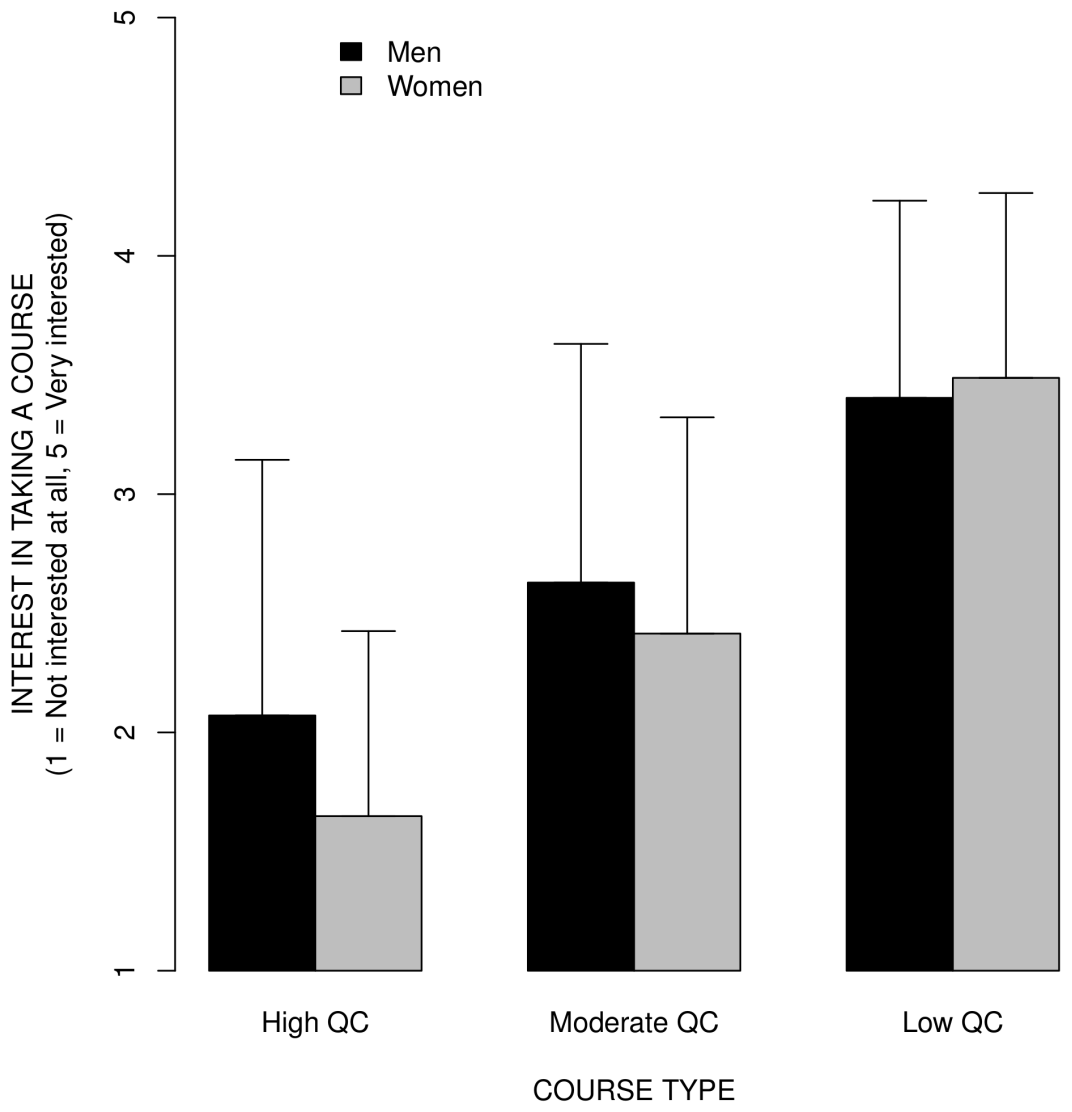
The mean course interest ratings for courses with high, moderate, and low QC, for women vs. men, for all participants. The error bars indicate ± 1 *SD* to allow visualization of the effect size. Women vs. men were much less interested in taking courses with high vs. low QC.

Finally, we examined whether women are less likely to be interested in courses with quantitative vs. non quantitative content than men. Because five participants did not reveal their sex, these analyses are based on 335 participants rather than 340 participants. Figure 5 shows the average course interest ratings for courses with high, moderate, and low QC, for women vs. men, with error bars indicating ± 1 *SD* to allow visualization of the effect size, for all participants. The figure highlights an interaction between sex and course type; women vs. men were much less interested in taking courses with high QC, less interested in taking courses with moderate QC, and about equally interested in taking courses with low QC. The ANOVA on participants' data with sex (men, women) as a between subject factor and course type (low QC, medium QC, high QC) as within subject factor revealed a significant main effect of sex, *F*(1,333) = 3.98, MSe = 1.49, *p* = 0.047; a significant main effect of course type, *F*(1,666) = 697.75, MSe = 0.36, *p* < .001; and a significant sex by course type interaction, *F*(1,666) = 10.45, MSe = 0.36, *p* < .001. Similarly, the ANOVA on the mean course ratings with course type (high QC, moderate QC, low QC) and sex (men, women) as between subject factors revealed a significant effect of course type, *F*(1,40) = 138.77, MSe = 0.152, *p* < .001 and a significant course type by sex interaction, *F*(1,40) = 17.88, MSe = 0.031, *p* < .001. However, the main effect of sex was not significant in this analysis, *F*(1,40) = 0.15, *Mse* = 0.031, *p* = .697. 

**Figure 6:**
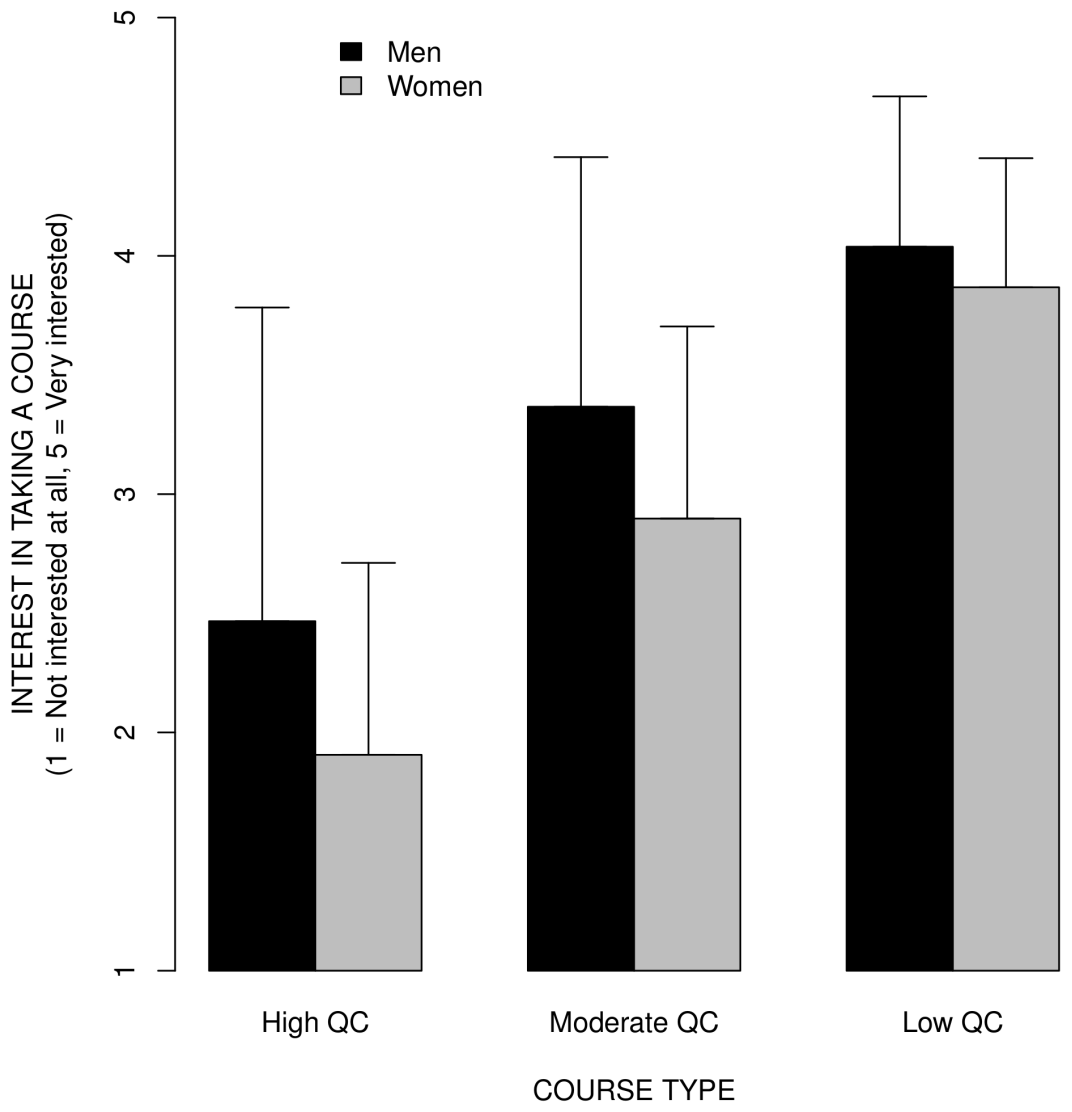
The mean course interest ratings for courses with high, moderate, and low QC, for women vs. men, for psychology majors only (10 men and 39 women). The error bars indicate ± 1 *SD* to allow visualization of the effect size. The pattern of results is similar to that found with all 335 participants; women vs. men were much less interested in taking courses with high vs. low QC.

Figure 6 shows the average course interest ratings for courses with high, moderate, and low QC, for women vs. men, with error bars indicating ± 1 *SD* to allow visualization of the effect size, for psychology majors only (10 men and 39 women). The pattern of results is similar to that found with all 335 participants who revealed their sex. However, the statistical analyses suffer from a low a priori statistical power due to a very small number of men in the psychology majors sample. The ANOVA on participants' data with sex (men, women) as a between subject factor and course type (low QC, medium QC, high QC) as within subject factor revealed only a significant main effect of course type, *F*(1,94) = 137.30, MSe = 0.316, *p* < .001. The main effect of sex was only marginally significant, *F*(1,47) = 3.00, MSe = 1.273, *p* = .090; and the sex by course type interaction was not significant, *F*(1,94) = 1.05, MSe = 0.316, *p* = .354. The ANOVA on the mean course ratings with course type (high QC, moderate QC, low QC) and sex (men, women) as between subject factors revealed a significant effect of course type, *F*(1,40) = 108.08, MSe = 0.208, *p* < .001; a significant effect of sex, *F*(1,40) = 23.15, MSe = 0.045, *p* =< .001; and a significant course type by sex interaction, *F*(1,40) = 9.20, MSe = 0.045, *p* = .004. 

## Discussion

Our findings show that undergraduate students have minimal interest in taking courses with any substantive quantitative content. The students were least interested in taking courses with high QC (statistics courses) and somewhat more interested in taking courses with moderate QC (research methods and research intensive courses). In terms of effect size, statistics courses were rated nearly 6 *SD* below the mean ratings of psychology content area courses and research methods courses were rated approximately 3 *SD* below the mean ratings of psychology content area courses. Out of 340 participants, fewer than 10 indicated that they were “very interested” in taking any of the three statistics courses. In contrast, nearly half (159 out of 340) were “very interested” in taking *Introduction to the Psychology of Abnormal Behavior* (269 out of 340, 79%, rated their interest in this course as 4 or 5 on the 5-point scale). Although psychology majors rated nearly all courses somewhat higher than non psychology majors, the pattern of findings summarized above did not differ between students majoring in psychology vs. other fields. Finally, women are less interested in taking quantitative vs. non quantitative courses than men even though women's and men's interest in taking non quantitative courses is similar.

Our findings are consistent with those of Hoyt and Perera [3] and extend them in several important ways. First, our participants were asked to rate their interest in courses they may take in the future whereas Hoyt and Perera [3] asked students to indicate their prior desire to take each course retrospectively, that is, after they nearly completed it. Second, we compared student interest in quantitative vs. non quantitative courses within a single discipline, psychology, whereas Hoyt and Perera [3] compared quantitative vs. non quantitative courses across several disciplines. Third, we quantified the magnitude of differences in student interest in taking quantitative vs. non quantitative courses. Hoyt and Perera [3] did not provide any effect sizes nor sufficient information to calculate the magnitude of interest effects. And fourth, we examined sex differences in student interest in quantitative vs. non-quantitative courses.

Our study was conducted in a medium size undergraduate university (with approximately 11,000 full time students) that offers only a BA degree in Psychology rather than a BSc degree. Accordingly, one may argue that our findings may be different if the study were conducted in a research-intensive university or perhaps in a university with a BSc in Psychology (rather than a BA). While this is a possibility, we think that the findings are unlikely to change substantially if the study were conducted elsewhere. First, Mount Royal has a selective admission process similar to those of research-intensive universities such as the University of Calgary. Second, students registered in BSc programs in other fields within Mount Royal rated their interest in quantitative vs. non quantitative courses similarly.

The results of our study have important implications. First, they suggest that using the same SET standards for faculty teaching quantitative vs. non quantitative courses is inappropriate and possibly discriminatory since faculty teaching quantitative courses, through no fault of theirs, find themselves facing students who do not want to be in their courses in the first place, and thus rate such courses lower than non quantitative courses regardless of who teaches them and how they teach them [3]. Accordingly, several researchers have argued that student interest in courses should be used to adjust SET ratings [3–6]. However, we are aware of only one SET program – the IDEA student ratings – that systematically adjusts SET ratings by students' prior interest in courses [3].

Second, if faculty teaching quantitative courses are held to the same SET standard as faculty teaching non quantitative courses, they are likely to feel pressure to dumb down their courses, for example, by selecting easy textbooks, providing exam questions in advance, allowing students to use formula sheets even for such basic concepts as mean and standard deviation, giving students step by step instructions on how to complete assignments using statistical software (e.g., open SPSS, click on FILE,...), avoiding harder topics such as statistical power, and in general, hold students to very low standards of performance. Students may be pleasantly surprised how easy such a statistics course can be, but will find themselves lacking the skills to analyze real-world datasets in subsequent advanced courses or in employment positions post-graduation. Such self-preserving teaching tactics are even more likely when institutions rely nearly exclusively on SETs to evaluate faculty teaching performance and when departmental chairs and administrators are unaware of the large differences in student' course interests and their impact on SETs. If administrators are not aware of these basic facts and do not know how to properly interpret SET ratings [10,11], what is a tenure track faculty member to do? They need to focus on SETs rather than on student learning for their own self-preservation and academic survival. Student learning and competence will necessarily take a back seat to the drive for higher SETs.

 Third, our findings have different policy implications depending on the balance of institutional focus on student satisfaction vs. student learning. At one end of the continuum, one could argue that institutions focused primarily on student satisfaction should eliminate courses with quantitative content such as statistics and research methods from their curriculum to increase overall student interest in their courses, student satisfaction, and student retention. At the other end of the continuum, institutions focused primarily on student learning and preparation for their future careers may want to abolish SETs altogether and focus on evaluation of student learning, using for example standardized common tests to evaluate program effectiveness. A middle ground may be to continue to require that students take quantitative courses as appropriate but that SETs are adjusted by student interest and contribute only a small percentage towards faculty teaching evaluations (e.g., 30%).

 Fourth, the lack of interest in quantitative and research methods courses among undergraduate students also threatens the very existence of psychology as well as other fields as a science [8]. Undergraduate students not interested in quantitative methods are unlikely to pursue doctoral degrees in quantitative psychology and unlikely to learn enough to be able to competently analyze their own data. Moreover, according to some experts, the shortage of faculty trained in quantitative methods is responsible for a frequent lack of quantitative skills among researchers and clinicians as well as for ”the dumbing down of quantitative understanding in psychological research” [1].

 Can we change students' attitudes towards quantitative courses such as statistics and research methods? In discussing what can be done about a related problem – lack of statistical understanding among patients, doctors, journalists as well as politicians, Gigerenzer, Gaissmaier, Kurt-Milcke, Schwartz, and Woloshin [12] argued that statistical thinking needs to be taught early in primary and secondary school using real world problems rather than dice and coins. Our data indicate that disinterest in quantitative courses is already present in students who have just entered university and are taking first year classes, before they have taken their first university statistics or research methods course. Accordingly, increasing students' exposure to mathematics and statistics using interesting real world problems rather than dice, coins, and beans early in primary and secondary education seems like an idea worth pursuing.

## References

[1] Clay R (2005) Too few in quantitative psychology: The subdiscipline sees shrinking numbers, but growing opportunities. Monit Psychol 36: 26.

[2] Hoyt DP, Lee E-J (2002) Basic data for the revised IDEA system. Manhattan, Kansas State University: IDEA Center.

[3] Hoyt DP, Perera S (2001) Are quantitatively-oriented courses different? Manhattan, KS: The IDEA Center

[4] Marsh HW, Dunkin M (1992) Students’ evaluations of university teaching: A multidimensional perspective. In: Smart JC Higher education: Handbook of theory and research, Vol. 8 New York: Agathon Press.

[5] Benton SL, Cashin WE (2012) Student ratings of teaching: A summary of research and literature. Manhattan, KS: The IDEA Center

[6] Cashin WE (1995) Student ratings of teaching: The research revisited. Manhattan, KS: Kansas State University, Center for Faculty Evaluation and Development.

[7] Lawson TJ, Reisinger DL, Jordan-Fleming MK (2012) Undergraduate psychology courses preferred by graduate programs. Teach Psychol 39: 181–184. doi:10.1177/0098628312450430.

[8] Aiken LS, Aguinis H, Appelbaum M, Boodoo GM, Edwards MC et al. (2007) Report of the task force for increasing the number of quantitative psychologists. American Psychological Association.

[9] Halpern DF, Benbow CP, Geary DC, Gur RC, Hyde JS et al. (2007) The Science of Sex Differences in Science and Mathematics. Psychol Sci Public Interest 8: 1–51. doi:10.1111/j.1529-1006.2007.00032.x. 25530726 PMC4270278

[10] Franklin J (2001) Interpreting the numbers: using a narrative to help others read student evaluations of your teaching accurately. New Dir Teach Learn 2001: 85–100 doi:10.1002/tl.10001.

[11] Wachtel HK (1998) Student evaluation of college teaching effectiveness: A brief review. Assess Eval HIGH Educ 23: 191–211. doi:10.1080/0260293980230207.

[12] Gigerenzer G, Gaissmaier W, Kurz-Milcke E, Schwartz LM, Woloshin S (2007) Helping doctors and patients make sense of health statistics. Psychol Sci Public Interest 8: 53 –96. doi:10.1111/j.1539-6053.2008.00033.x. 26161749

